# Identification and Regulation of c-Myb Target Genes in MCF-7 Cells

**DOI:** 10.1186/1471-2407-11-30

**Published:** 2011-01-25

**Authors:** Anita M Quintana, Fan Liu, John P O'Rourke, Scott A Ness

**Affiliations:** 1 Department of Molecular Genetics and Microbiology, University of New Mexico Health Sciences Center, Albuquerque, NM 87131-0001 USA; 2 St. Jude Children's Research Hospital, Memphis, TN 38105-2794, USA; 3 Sloan-Kettering Institute, Memorial Sloan-Kettering Cancer Center, New York, NY 10021, USA

## Abstract

**Background:**

The c-Myb transcription factor regulates differentiation and proliferation in hematopoietic cells, stem cells and epithelial cells. Although oncogenic versions of c-Myb were first associated with leukemias, over expression or rearrangement of the c-*myb *gene is common in several types of solid tumors, including breast cancers. Expression of the c-*myb *gene in human breast cancer cells is dependent on estrogen stimulation, but little is known about the activities of the c-Myb protein or what genes it regulates in estrogen-stimulated cells.

**Methods:**

We used chromatin immunoprecipitation coupled with whole genome promoter tiling microarrays to identify endogenous c-Myb target genes in human MCF-7 breast cancer cells and characterized the activity of c-Myb at a panel of target genes during different stages of estrogen deprivation and stimulation.

**Results:**

By using different antibodies and different growth conditions, the c-Myb protein was found associated with over 10,000 promoters in MCF-7 cells, including many genes that encode cell cycle regulators or transcription factors and more than 60 genes that encode microRNAs. Several previously identified c-Myb target genes were identified, including CCNB1, MYC and CXCR4 and novel targets such as JUN, KLF4, NANOG and SND1. By studying a panel of these targets to validate the results, we found that estradiol stimulation triggered the association of c-Myb with promoters and that association correlated with increased target gene expression. We studied one target gene, CXCR4, in detail, showing that c-Myb associated with the CXCR4 gene promoter and activated a CXCR4 reporter gene in transfection assays.

**Conclusions:**

Our results show that c-Myb associates with a surprisingly large number of promoters in human cells. The results also suggest that estradiol stimulation leads to large-scale, genome-wide changes in c-Myb activity and subsequent changes in gene expression in human breast cancer cells.

## Background

The importance of the c-Myb transcription factor in breast cancer is closely linked to the response to estrogen [1]. Expression of the c-*myb *(MYB) gene is associated with expression of estrogen receptors (ERs) in breast tumors [2,3]. (Note: We use c-Myb and c-*myb *to distinguish between the protein and gene, respectively.) Regulation by ERs has been implicated in the post-transcriptional regulation of c-*myb *gene expression [4] and the c-*myb *gene is involved in recurrent translocations in some breast tumors that are positive for expression of ERs [5]. The c-*myb *gene is induced by activation of ERs in breast cancer cell lines such as MCF-7 [6-8] and c-Myb protein has been implicated in the regulation of several genes important in breast cancer development and progression, including BRCA1 [9], CXCL12 [10], Mdm2 and p53 [11]. Although the expression of c-Myb protein is important for estrogen-stimulated proliferation of breast cancer cells [6], the functions of c-Myb and the target genes that it regulates in response to stimulation of ERs have yet to be identified.

The c-*myb *gene is a cellular proto-oncogene from which the v-*myb *oncogenes expressed by two avian leukemia viruses are derived [12]. The v-*myb *oncogenes transform hematopoietic cells in tissue culture and induce leukemias in animals, and a mouse knockout of c-*myb *leads to severe hematopoietic defects [13], which has led many researchers to focus on the role of the Myb proteins in hematopoietic cells. However, increasing evidence has demonstrated an important role for c-Myb expression in several epithelial cell types, including breast and colon [14,15] and there are examples where activated or rearranged alleles of c-*myb *play important roles in epithelial tumors [14-17]. Since the c-Myb protein is a DNA-binding transcription factor, its oncogenic activity is likely linked to its ability to regulate specific target genes that affect cell proliferation or tumorigenesis. Microarray studies have proved to be a powerful tool for studying the activities of Myb proteins, and they have identified dozens of genes that are induced when c-Myb is ectopically over-expressed in MCF-7 breast cancer cells and other cell types [18,19]. However, it is not clear whether those genes are directly or indirectly regulated by c-Myb, whether they are also regulated by c-Myb expressed at its normal levels, or how their regulation is affected by stimulation of ERs or other extra-cellular stimuli.

Chromatin immunoprecipitation (ChIP) offers an approach for using specific antibodies to enrich for fragments of the genome associated with a specific protein, e.g. a transcription factor [20]. ChIP has proved to be extremely powerful for studying epigenetic modifications of genes undergoing activation or silencing or for confirming that specific transcription factors like c-Myb associate with promoters in cells [10]. When the ChIP samples are analyzed on a microarray of probes specific for thousands of promoter regions (ChIP-on-chip), or directly identified by sequencing the recovered DNA fragments (ChIP-seq), the results can be used to map the locations of transcription factor binding sites across the whole genome [21]. However, one of the surprises of such studies is the large number of promoters or binding sites that have been identified. For example, recent studies have shown that the transcription factor Sall4 associates with 3,223 genes [22], c-Myc associates with more than 4,000 genes [23], estrogen receptor alpha associates with more than 10,000 genes [24] and NFkappaB associates with more than 15,000 genes [25]. In this manuscript, we describe the use of ChIP-on-chip approaches to map more than 10,000 binding sites for c-Myb in MCF-7 cells. The collection includes nearly all previously identified c-Myb target genes plus many new targets, and the results have important implications for how c-Myb regulates the proliferation of breast cancer cells and how it participates in tumorigenesis.

## Methods

### Cells and culture conditions

MCF-7 cells (ATCC, Manassas, VA) were cultured at 37°C/5% CO_2 _in Dulbecco's Modified Eagle's Medium (DMEM; Invitrogen, Carlsbad, CA) supplemented with 10% (v/v) fetal bovine serum (Invitrogen, Carlsbad, CA). For estrogen deprivation, cells (5×10^5 ^per/ml) were cultured in phenol red free DMEM (Invitrogen, Carlsbad, CA) supplemented with 5% charcoal stripped serum (Invitrogen, Carlsbad, CA) for 48 hr. The deprived cells were then stimulated by adding 10 nM beta-estradiol (Sigma, St. Louis MO) for 24 hr. Changes in DNA synthesis rates (data not shown) were used to verify that the MCF-7 cells arrested and resumed growth under these conditions, as reported previously [6].

### Expression analysis, transcription assays, and immunoprecipitation

Steady state RNA expression analysis, quantitative real-time PCR (QPCR), transcriptional assays, immunoprecipitation, and Western blots were performed as described previously [18,26,27]. Primer pairs and Taqman probes are listed in Additional file 1: Table S1 and Additional file 1: Table S2. The CXCR4 reporter gene was generated by cloning the region upstream of the CXCR4 start site into the pGL2 basic vector (Invitrogen, Carlsbad, CA). For isolation of cytoplasmic and nuclear fractions, MCF-7 cells were harvested and resuspended in 5 pellet volumes (500 μl) of cell lysis buffer (10 mM Tris-HCl pH 8.0, 85 mM KCl, 0.5% NP-40 plus protease inhibitors: 1 μM each chymostatin, leupeptin, antipain, pepstatin-A; 1 mM each phenylmethylsulfonyl fluoride and benzamidine) and incubated on ice for 10 min. Lysates were passed twice through a 26G 5/8" needle. Nuclei were collected by centrifugation at 10,000 × g for 5 min then lysed in 500 μl of nuclear lysis buffer (50 mM Tris pH 8.0, 1% SDS, 10 mM EDTA, plus protease inhibitors as described above). Under these conditions, nuclear proteins such as histones were found only in the nuclear fractions, while control cytoplasmic proteins such as GAPDH were only in the cytoplasmic fraction (data not shown). Protein concentrations were determined using Dc protein reagent (BioRad, Hercules, CA) according to manufacturer's instructions. Western blots were developed using rabbit antiserum raised against a bacterially expressed fragment of the c-Myb DNA binding domain [28].

### Chromatin immunoprecipitation (ChIP) assays

A detailed protocol for the ChIP and ChIP-chip assays is provided in the supplemental methods (see Additional file 1). Briefly, ChIP assays were performed using standard methods [20] but shearing was performed in a 200 μl volume of 50 mM Tris pH 8.0 (Sigma, St. Louis, MO) by adding 40 units of Micrococcal nuclease (USB, Cleveland, Ohio) for 10 min at 37°C. EDTA (10 mM) was used to stop the reaction and nuclei were lysed in 1% SDS. ChIP was performed with anti-Myb monoclonal 1.1 antibodies (Millipore, Billerica MA), control non-immune serum, anti-FLAG (Sigma, St. Louis MO) or with a rabbit anti-peptide antiserum prepared by using a peptide (HQGTILDNVKNLLEFAE) from the c-Myb transcriptional activation domain as antigen (Ab 1493). Immunoprecipitates were collected, washed and de-crosslinked, and the DNA was purified using standard methods [20]. Primers used for QPCR reactions using SYBR green (Biorad, Hercules CA) are listed in Additional file 1: Table S3. ChIP assay results were normalized for control genes (GAPDH) and control non-immune antibodies. For ChIP-on-chip assays, DNA (10 ng) was amplified according to manufacturer's protocol (http://www.affymetrix.com), but conditions were controlled carefully to insure that antibody-specific enrichment was preserved. Amplified DNA (6 μg) was hybridized to Affymetrix promoter tiling array 1.0R and data analysis was performed with Model-based Analysis for Tiling (MAT) Arrays software using the P value of 1×10^-5 ^recommended by the authors [29]. Statistical analysis was performed with PASW 17 (SPSS Inc., Chicago Ill) software. Data were visualized in Integrated Genome Browser (http://www.affymetrix.com). The ChIP-on-chip data files have been deposited in the NCBI GEO database (accession number: GSE18706).

### Myb knockdown

Knockdown of c-*myb *was performed using an shRNA expression vector kindly provided by Dr. Tom Gonda [6]. Briefly, cells were transduced with a lentivirus expressing a doxycycline-inducible shRNA specific to c-*myb *or a scrambled shRNA control. Expression of each shRNA was induced for 24 hr with 5 μg/ml of doxycycline and RNA levels were analyzed by QPCR as described above.

### Myb lentivirus production

N-terminal FLAG-tagged human c-Myb [18] was cloned into the unique PacI site of the pHR IRES GFP lentiviral vector (kindly provided with packaging vectors by Dr. Bruce Bunnell, Tulane University) downstream of an EF1 alpha promoter. v-Myb was cloned in a similar fashion except into the pLenti-6 vector (Invitrogen, Carlsbad CA). Plasmids were transfected into 293 FT cells (Invitrogen, Carlsbad CA) by calcium phosphate transfection along with the lentiviral packaging plasmid delta 8.9 and the pMD.G plasmid expressing the vesicular stomatitis virus glycoprotein. Supernatant was collected at 24 hr and 48 hr. Ultrafiltration using an Ambion Ultracell 100 kDa NMWL filter unit (Millipore, Billerica MA) was used to concentrate viral supernatants. Cells were transduced in the presence of 8 μg/ml of polybrene (Sigma, St. Louis MO) and sorted to enrich for GFP+ transduced cells or selected with 5 μg/ml blasticidin (Sigma, St. Louis, MO).

## Results

### c-myb expression is estrogen dependent

**Figure 1:**
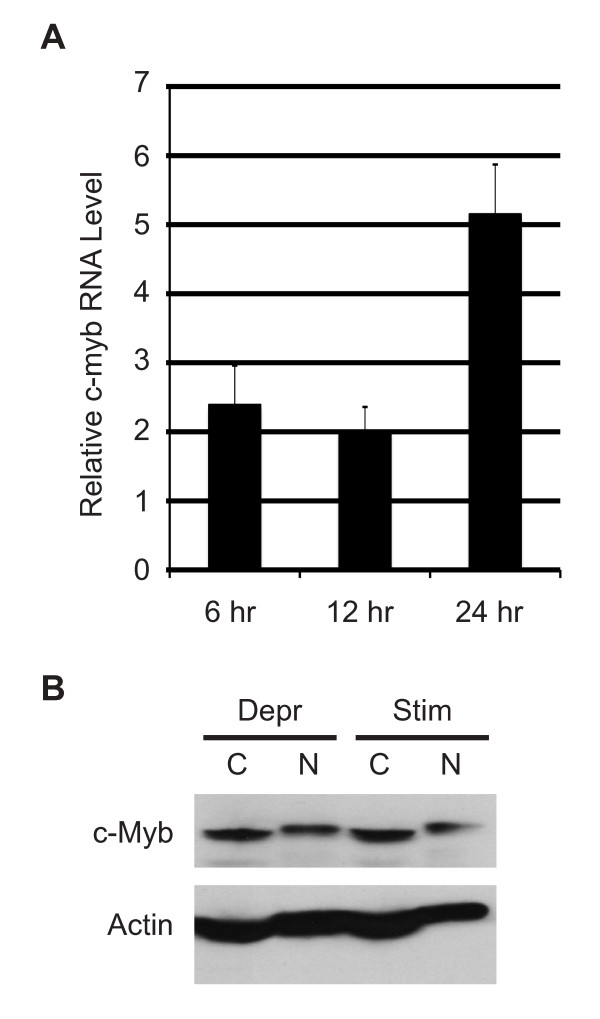
**c-Myb expression is estrogen regulated**. (A) MCF-7 cells were deprived of estrogen for 48 hr then stimulated with 10 nM 17-beta-estradiol for 6, 12 or 24 hr. Quantitative real time PCR (QPCR) was used to measure the levels of *c-myb *RNA during the deprivation and stimulation protocol. Error bars show standard deviation in triplicate PCR reactions, and results are relative to the estrogen-deprived cells. (B) Western blot with anti-c-Myb and anti-beta-actin antibodies of estrogen-deprived (Depr) or stimulated (Stim) MCF-7 cells, divided into cytoplasmic (C) or nuclear (N) fractions as described in Methods.

Several published studies suggest that c-*myb *RNA expression is tightly regulated by estrogen and tamoxifen in breast cancer cells [4,6,7], but the regulation of c-Myb protein levels has been less well characterized. To establish a link between the activation of estrogen receptors and the regulation of c-Myb protein levels and activity, we arrested MCF-7 cells by first depriving them of estrogen, then stimulating them with beta-estradiol as previously described [6]. In a time-course experiment, c-*myb *RNA levels increased about 2-fold relative to the estrogen-deprived cells by 6 hr (Figure 1B) and increased to about 5-fold by 24 hr. Thus, c-*myb *RNA levels were induced by beta-estradiol stimulation in MCF-7 cells. These results agree very nicely with similar results published by others [4,6,7].

However, we got different results when checking the expression of c-Myb protein. As shown in Figure 1B, there was little to no change in c-Myb protein levels in cells that were deprived (Depr) by growing them in estrogen-free medium or stimulated (Stim) for 24 hr with beta-estradiol. In addition, similar amounts of c-Myb protein were recovered in the crude cytoplasmic (C) and nuclear (N) extracts isolated from the two cell types. This result is different than hematopoietic cells, in which c-Myb protein is largely or exclusively in the nucleus [30]. Time course experiments showed little or no difference in c-Myb protein levels in any of the time points (similar results were obtained with multiple anti-c-Myb antibodies, data not shown). This suggests that the steady-state level of c-Myb protein remains fairly constant in MCF-7 cells, and also that c-Myb does not undergo gross changes in sub-cellular localization during or after estrogen stimulation, despite the increased levels of c-*myb *RNA. Thus, if increased levels of c-*myb *mRNA lead to increased synthesis, it must be balanced by faster turnover of c-Myb protein in the estrogen-stimulated cells. We conclude that c-Myb protein is present at similar and fairly constant levels in both estrogen-deprived and stimulated MCF-7 cells, raising the possibility that it could regulate different sets of target genes in the two conditions.

### Identification of c-Myb target genes in MCF-7 cells

**Figure 2:**
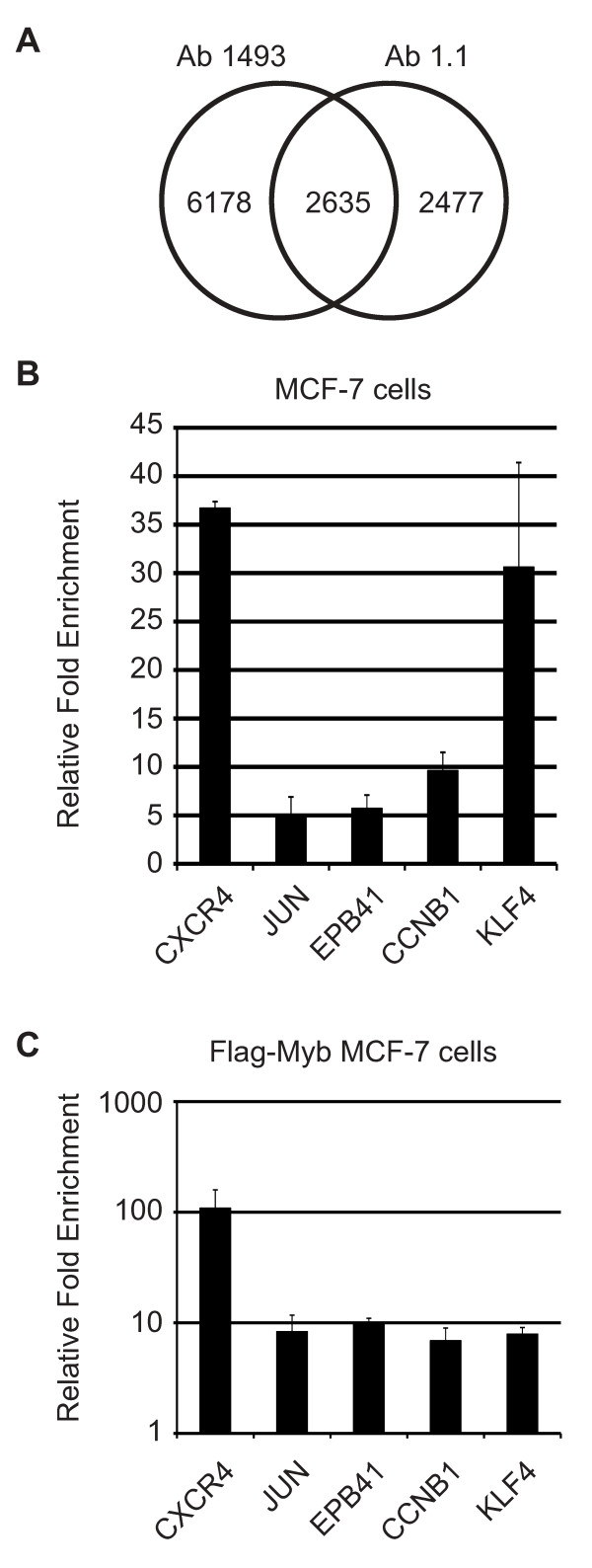
**ChIP on chip identifies c-Myb target genes**. (A) ChIP-on-chip was performed with anti-Myb antibodies 1493 and 1.1 using chromatin from MCF-7 cells that were deprived of estrogen for 48 hr followed by 24 hr of estrogen stimulation or grown to high density. The Venn diagram summarizes the statistically significant (P = 1×10^-5^) binding sites identified by each antibody in at least one of the two growth conditions. The complete list of binding sites is provided in Additional file 2, Additional file 3, Additional file 4, Additional file 5 and Additional file 6. (B) ChIP with anti-c-Myb antibodies. MCF-7 cells were subjected to chromatin immunoprecipitation with anti-c-Myb antibodies. Enrichment for the CXCR4, JUN, EPB41, CCNB1, and KLF4 promoters was assessed by QPCR. Fold enrichment was calculated relative to a control gene (GAPDH) and a control antibody (mouse non-specific IgG). Error bars show standard deviation of triplicate PCR reactions. (C) Conventional ChIP with anti-FLAG antibodies was performed on MCF-7 cells transduced with FLAG epitope-tagged c-Myb, using anti-FLAG antibodies. Fold enrichment was calculated as described above.

Microarray experiments have shown that overexpression of the normal c-Myb protein or the oncogenic derivative v-Myb protein from Avian Myeloblastosis Virus in MCF-7 cells leads to the activation of different sets of target genes [18] and that c-Myb expression is necessary for beta-estradiol-induced proliferation of MCF-7 cells [6]. We turned to genome-wide Chromatin Immunoprecipitation (ChIP) coupled with promoter microarrays, or ChIP-on-chip assays, to identify c-Myb target genes that could be involved in regulating the proliferation of MCF-7 cells. This approach had the advantage of following endogenous genes and did not depend on the over-expression of c-Myb, which could affect c-Myb specificity and activity. Target genes regulated by c-Myb have been identified in a variety of different cell types and culture conditions and the expression of the c-*myb *gene is regulated by estrogen in MCF-7 cells, although there is some evidence that c-*myb *gene regulation may be due to non-genomic pathways that do not involve the classical estrogen receptors [31]. Therefore, we set up our experiments to be as inclusive as possible and included cells that had been estrogen deprived and then stimulated with beta-estradiol for 24 hr as well as cells that were grown to high density in complete medium. Briefly, MCF-7 cells grown under the two conditions were treated with formaldehyde to cross-link DNA-protein complexes. After purification, fragmented chromatin complexes were immunoprecipitated with two different anti-Myb antibodies, (1493 or Ab1.1, also referred to as VKN or Upstate antibodies, respectively) or with a control IgG antibody. Purified DNA was amplified, labeled and hybridized to Affymetrix Promoter Tiling arrays with probes spanning 10 kb regions around the promoters of approximately 25,000 known human genes. This approach identified a combined 8,813 target genes with the first antibody in the two different growth conditions, and combined 5,112 targets with the second antibody in the two conditions, for a total of 11,290 statistically significant (P = 1×10^-5^) binding sites enriched by one or more of the Myb antibodies in one or both of the growth conditions in MCF-7 cells (Figure 2A). This P value was determined empirically, based on recommendations by the authors of the analysis software [29] and is intended to be inclusive in order to detect as many Myb binding sites as possible. Analysis of the enriched DNA sequences showed that approximately 75% of the sites contained predicted c-Myb binding sites (data not shown). The identified genes included previously described c-Myb targets BRCA1, CCNB1, CCNE1, CXCR4, KIT, MYB and MYC and other genes that have not been previously identified as Myb-regulated genes, including ELK4, EPB41, JUN, KLF4, NANOG and SND1, as well as more than 60 genes that encode microRNAs (see Additional file 1: Figure S1). A core group of 2,635 target genes were identified by both antibodies in one or both of the growth conditions.

### ChIP and QPCR validate ChIP-on-chip results

We chose a panel of 5 genes to validate some of the results from the ChIP-on-chip assays. Our laboratory previously identified the CXCR4 gene as a potential Myb target gene after it was up-regulated in MCF-7 cells expressing the v-Myb or c-Myb transcription factors [18]. JUN encodes a component of the transcription factor AP-1, and is, like c-Myb, a proto-oncogene. EPB41 encodes the erythrocyte protein band 4.1, which was first identified in erythroid cells but is also expressed on other normal and cancer cell types [32,33]. CCNB1 encodes the cell cycle regulator Cyclin B1 and has been previously identified as a c-Myb target gene in hematopoietic cells [34] and KLF4 encodes the zinc finger transcription factor Kruppel-like factor 4, which is important in the regulation of hematopoiesis and in the production of induced pluripotent stem cells [35,36].

We used a conventional ChIP assay, followed by Quantitative real-time PCR (QPCR) with primers from the regions identified in the promoter arrays, to validate the c-Myb binding sites in these selected genes. As shown in Figure 2B, the promoters of the CXCR4 and KLF4 genes were enriched approximately 35-fold and 30-fold by the Myb antibodies, respectively. The JUN, EPB41, and CCNB1 binding sites were enriched 5 to 10 fold compared to the control gene (GAPDH) or the background detected using the control non-specific immunoglobulin (IgG). In addition to these five genes, we also tested 10 additional randomly chosen genes identified in the ChIP-on-chip assays, and all but one was enriched and validated in the conventional ChIP assay (data not shown). Based on these results, we conclude that most of the binding sites identified in the ChIP-on-chip assays are likely to be bona fide c-Myb targets, suggesting that c-Myb occupies thousands of promoters in MCF-7 cells. Details about all of the binding sites we identified are provided in the supplemental materials (see Additional file 2, Additional file 3, Additional file 4, Additional file 5 and Additional file 6) and the complete data sets have been deposited in the NCBI GEO database (accession number: GSE18706).

In addition to c-Myb, MCF-7 cells also express several Myb-related proteins, including A-Myb (MYBL1) and B-Myb (MYBL2), which are also induced following beta-estradiol treatment [7]. Although we used two different antibodies that should be specific for c-Myb, there was a possibility that we had detected one or both of the other Myb proteins in our assays. As an extra level of validation, we transduced MCF-7 cells with a lentivirus expressing FLAG epitope-tagged c-Myb and used anti-FLAG antibodies in the ChIP assay protocol. As shown in Figure 2C, ChIP assays using anti-FLAG antibodies enriched for the CXCR4 promoter approximately 100-fold, and for the JUN, EPB41, CCNB1, and KLF4 promoters about 10-fold. Control genes (GAPDH) and other regions of the CXCR4 promoter that do not contain c-Myb binding sites were not enriched in these assays (data not shown). We conclude that the ChIP assays are specific and identify bona fide c-Myb targets in MCF-7 cells.

### c-Myb binding specificity is affected by beta-estradiol treatment

**Figure 3:**
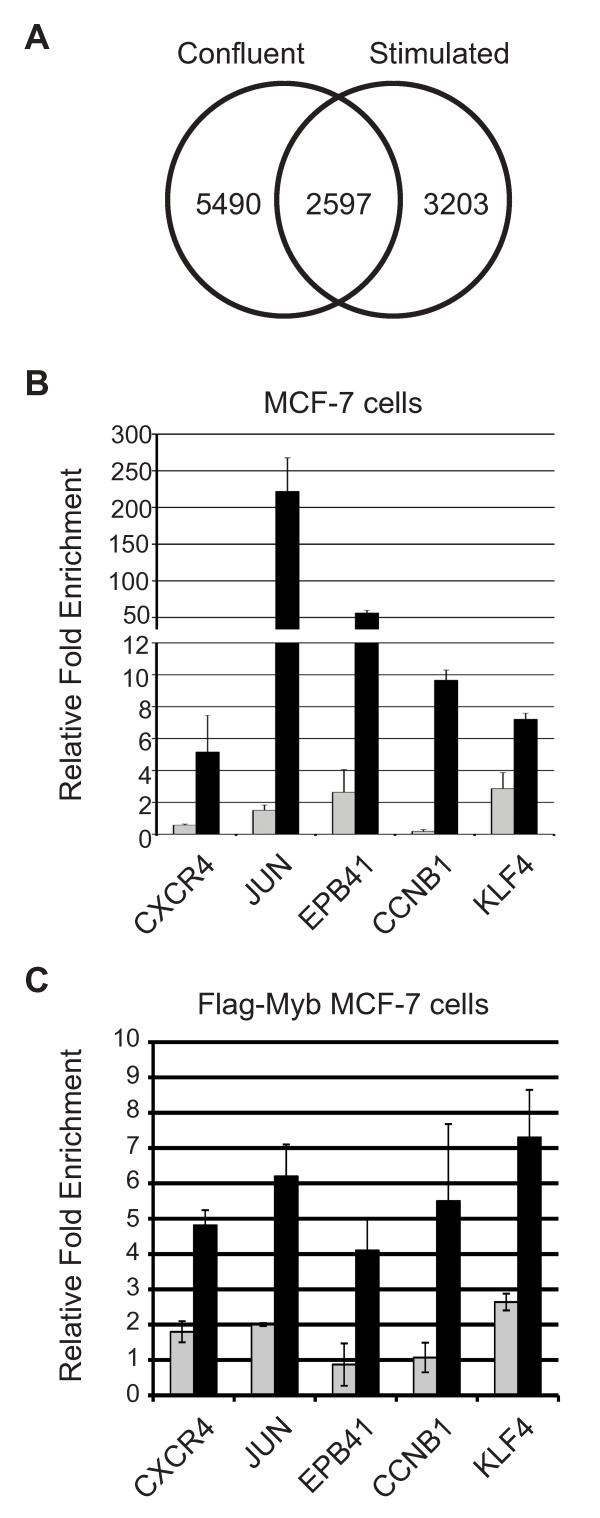
**c-Myb activity is estrogen stimulated**. (A) Comparison of ChIP-on-chip results obtained with MCF-7 cells that were deprived of estrogen for 48 hr followed by 24 hr of estrogen stimulation (Stimulated) or grown to high density (Confluent). The Venn diagram summarizes the statistically significant (P = 1×10^-5^) binding sites identified in the two growth conditions. The complete list of binding sites is provided in Additional file 2, Additional file 3, Additional file 4, Additional file 5 and Additional file 6. (B) ChIP was performed on MCF-7 cells deprived of estrogen for 48 hr (gray bars) or deprived and then stimulated with 10 nM 17-beta-estradiol for 24 hr (black bars). Chromatin complexes were immunoprecipitated with anti-c-Myb 1.1 antibodies. Enrichment for the CXCR4, JUN, EPB41, CCNB1, and KLF4 promoters were measured by QPCR. Error Bars represent standard deviation of triplicate PCR reactions. (C) ChIP was performed as in (B) using MCF-7 cells expressing FLAG-tagged c-Myb and chromatin complexes were immunoprecipitated with anti-FLAG antibodies. Enrichment for the CXCR4, JUN, EPB41, CCNB1, and KLF4 promoters were measured by QPCR. Error Bars represent standard deviation of triplicate PCR reactions.

We also analyzed our ChIP-on-chip data to see if c-Myb associated with the same promoters in the two growth conditions, confluent cells vs. cells that had been estrogen-deprived then stimulated with beta-estradiol. As summarized in Figure 3A, c-Myb was bound to 5,490 promoters in the confluent cells, 3,203 promoters in the stimulated cells, and 2,597 promoters in both conditions (these numbers are derived by pooling the results obtained with the two antibodies in the two different growth conditions, which were analyzed separately). The ChiP-on-chip results suggest that a dramatic relocalization of c-Myb occurs, with the protein bound to largely different sets of target genes in the two growth conditions.

We used the panel of genes we identified in Figure 2 to characterize changes in c-Myb specificity during estrogen depletion or stimulation with beta-estradiol. As shown in Figure 3B we did not detect significant levels of c-Myb at any of the promoters during estrogen deprivation, although c-Myb protein was abundantly expressed (Figure 1). Upon beta-estradiol stimulation we observed a marked increase of c-Myb binding at the JUN and EPB41 promoters (Figure 3B) and a smaller, but still significant 5 to 10-fold enrichment of the CXCR4, CCNB1, and KLF4 gene promoters. Thus, although c-Myb protein was expressed equally during estrogen deprivation and stimulation (Figure 1), it was only detectable at the target gene promoters in the latter cells. To extend our results we repeated this experiment using the MCF-7 cells expressing FLAG-tagged c-Myb. Western blot analysis demonstrated that FLAG-tagged c-Myb was expressed in transduced cells (see Additional file 1: Figure S2). As shown in Figure 3C, estrogen stimulation also led to an increased enrichment of all the target gene promoters when anti-FLAG antibodies were used for the ChIP assays.

These results are somewhat paradoxical. The Western blot (Figure 1B) showed that c-Myb protein is stably expressed during estrogen starvation, but the ChIP assays were unable to detect c-Myb bound to any of the target genes we tested under those conditions. In contrast, c-Myb association with the promoters greatly increased following beta-estradiol stimulation. The results suggest that stimulation leads to large changes in the activity of c-Myb and its ability to associate with specific target gene promoters. Our analysis showed that only about 30% of the identified c-Myb binding sites also contained potential sites for the binding of ERs (data not shown), suggesting that the mechanism affecting changes in c-Myb activity may be due to non-genomic effects or the activation of signaling pathways that lead to changes in c-Myb protein activity or targeting. Interestingly, ERalpha has been shown to be important in the regulation of c-*myb *gene expression [6], suggesting that multiple estrogen-responsive pathways contribute to the regulation of c-Myb protein levels and its activity.

### A disconnect between c-Myb binding and gene activation

**Figure 4:**
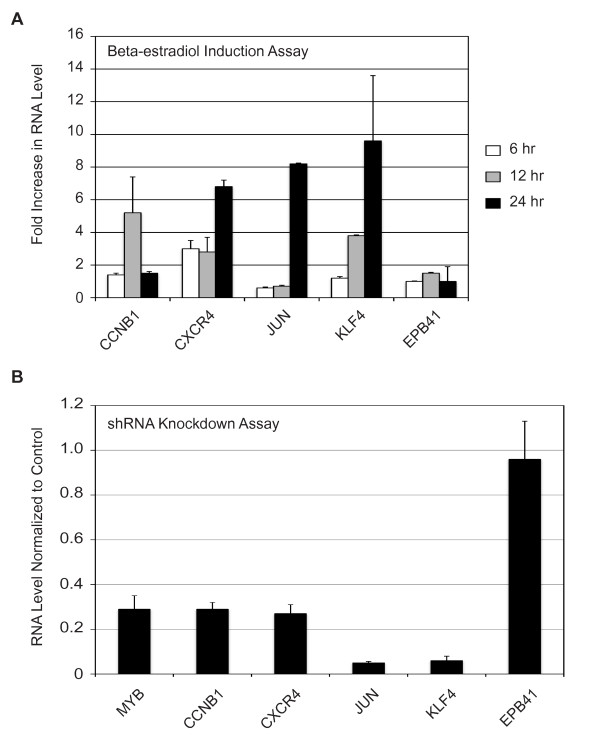
**Estrogen changes c-Myb activity**. (A) MCF-7 cells were deprived of estrogen for 48 hr then stimulated with 10 nM 17-beta-estradiol for 6, 12 or 24 hours. QPCR was used to measure the levels of CCNB1, CXCR4, JUN, KLF4 and EPB41 RNAs. Error bars show standard deviation in triplicate PCR reactions, and results are relative to the estrogen-deprived cells. (B) MCF-7 cells were transduced with retroviral vectors expressing doxycycline inducible shRNAs (scrambled or c-*myb *specific). Each cell line was induced for 24 hr with doxycycline and the relative expression of c-*myb*, CCNB1, CXCR4, JUN, KLF4 and EPB41 RNAs was measured by QPCR. Data are normalized to the scrambled control shRNA.

To address the impact of c-Myb binding to the target gene promoters, we performed a time course experiment with cells deprived of estrogen or treated with beta-estradiol for up to 24 hr and analyzed the relative expression of each of the genes characterized above. Interestingly, each of the target genes described above displayed a different pattern of expression in the time course experiment. Compared to the estrogen-deprived cells, the CCNB1 gene showed no response at 6 hr but was about 5-fold induced at 12 hr and returned to baseline expression by 24 hr (Figure 4A). CXCR4 was about 3-fold induced at 6 and at 12 hr, but was nearly 7-fold induced by 24 hr. JUN showed no induction at 6 or 12 hr, but was strongly activated at 24 hr. The KLF4 gene showed no response at 6 hr, but was induced 4-fold by 12 hr and more than 8-fold at 24 hr. Only the EPB41 gene failed to be activated following beta-estradiol stimulation. Although c-Myb was bound to each of these target promoters after addition of beta-estradiol, the genes responded quite differently, suggesting that, at least for some genes, changes in the binding of c-Myb to the promoters was not sufficient to cause increased expression.

Because the kinetics of activation of the genes was so different, we used an shRNA knock down strategy to test whether c-Myb was required for their expression. We introduced a lentivirus harboring a doxycycline-inducible shRNA [6] targeting the c-*myb *mRNA (kindly provided by T. Gonda) into the MCF-7 cells, then tested the effects of doxycycline-induced knock down of c-Myb. The shRNA construct efficiently knocked down c-Myb protein expression after 24 hr of doxycycline treatment relative to the scrambled control (see Additional file 1: Figure S2) and c-*myb *RNA levels decreased approximately 70% (Figure 4B). Levels of the CCNB1, CXCR4, JUN and KLF4 target gene mRNAs were also dramatically reduced by the shRNA targeting c-*myb *(Figure 4B). The EPB41 RNA, which was not inducible by beta-estradiol (panel A), was also not affected by the c-*myb *shRNA. The scrambled control shRNA did not lead to significant decreases in any of the RNAs. These results confirm that c-Myb expression is required to maintain expression of the CCNB1, CXCR4, JUN and KLF4 genes in MCF-7 cells, suggesting that these genes are endogenous targets of c-Myb. The EPB41 gene has a c-Myb binding site, but its expression is not significantly regulated by estrogen deprivation or beta-estradiol stimulation or dependent on c-Myb in these cells.

### Binding sites for c-Myb in the CXCR4 promoter

To further confirm the ChIP results, we decided to investigate the regulation of one c-Myb target gene in more detail. CXCR4 is the receptor for the chemokine SDF-1, is important in the metastatic potential of estrogen receptor positive ovarian and breast cancer [37,38] and is linked to poor prognosis in a variety of tumor types and leukemias [39-41]. We previously identified CXCR4 as a potential c-Myb target gene in microarray experiments using MCF-7 cells [18,19]. The promoter of the CXCR4 gene has been characterized, but was not described as having binding sites for c-Myb [42]. However, there are at least 10 potential c-Myb binding sites in the CXCR4 promoter, although some contain mismatches from the normal consensus (see Additional file 1: Table S4). Since our ChIP results showed that c-Myb binds the CXCR4 promoter in human cells, we decided to characterize the interactions more directly.

**Figure 5:**
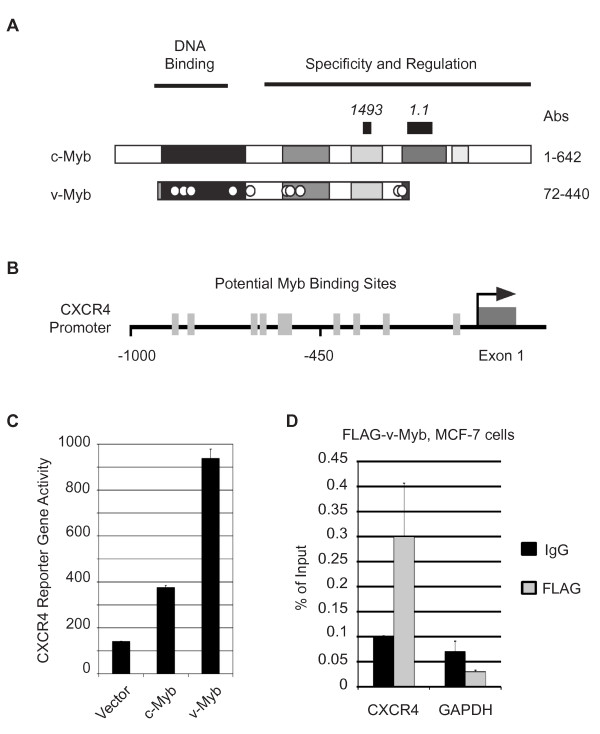
**The *CXCR4 *promoter is regulated by Myb proteins**. (A) Structures of c-Myb and v-Myb proteins. The diagrams depict the structures of the c-Myb and v-Myb proteins, which share conserved domains (shaded) involved in DNA binding and regulation. The oncogenic v-Myb protein is truncated at both ends and has a number of point mutations represented by white dots. The locations of the epitopes for antibodies (Abs) 1493 and 1.1 are indicated. (B) Structure of the CXCR4 gene promoter. The region upstream of the human CXCR4 gene is diagrammed, with putative Myb binding sites indicated by gray boxes. The arrow indicates the start site and direction of transcription. (C) Activation of a CXCR4 reporter gene. A reporter construct containing the CXCR4 promoter upstream of the luciferase reporter gene was co-transfected into HEK293 cells along with control plasmid (vector) or plasmids expressing c-Myb or v-Myb, as indicated. The figure shows reporter gene activity. Error bars show standard deviation of triplicate assays. (D) MCF-7 cells were transduced with a lentivirus expressing FLAG-tagged v-Myb. ChIP was performed with anti-FLAG antibodies (gray Bars) or control IgG (black bars). QPCR was performed with primers specific to CXCR4 and GAPDH. Data is normalized relative to percent of input. Error bars represent standard deviation of triplicate PCR reactions.

Previous work from our laboratory [18] showed that CXCR4 gene expression increased substantially following ectopic expression of either c-Myb or its oncogenic derivative v-Myb (Figure 5A). We isolated and analyzed the human CXCR4 gene promoter and used it to assemble a luciferase-based reporter gene construct (Figure 5B). As shown in Figure 5C, both c-Myb and v-Myb activated the reporter gene when they were co-transfected into HEK293 cells (which have very low endogenous c-Myb levels) but v-Myb had more activity at the CXCR4 promoter. These results suggest that both c-Myb and v-Myb regulate the expression of the CXCR4 gene by directly binding its promoter. Finally, to confirm that v-Myb also interacted with the endogenous CXCR4 promoter in MCF-7 cells, we generated MCF-7 cells expressing a FLAG epitope-tagged version of v-Myb (Additional file 1: Figure S2), then did a ChIP assay using anti-FLAG antibodies as described in the previous sections. As shown in Figure 5D, the FLAG-tagged v-Myb bound to the CXCR4 promoter, but not to an endogenous control promoter, GAPDH. Taken together, these results confirm that the CXCR4 gene is regulated by both normal and oncogenic variants of c-Myb in human cells.

## Discussion

### c-Myb binds to thousands of endogenous target genes

We performed a genome wide analysis to identify c-Myb target genes and although we demonstrated that c-Myb is bound to a substantial fraction of the genes, our numbers are consistent with other similar studies. ChIP-on-chip or ChIP followed by high throughput sequencing (ChIP-seq) has been performed with a variety of transcription factors such as c-Myc, Sall4, estrogen receptor alpha and NFkappaB [22,24,25,43]. Genome wide analyses identified approximately 8,000, 3,200, 10,000 and 15,000 binding sites for these four transcription factors, respectively. The results suggest that many transcription factors, including c-Myb, are bound to large and diverse sets of target genes. To validate our ChIP-on-chip results, we analyzed 15 of the identified binding sites using conventional ChIP assays, and were able to confirm the association with c-Myb for 14 (>90%) of them. However, the expression of at least some of the identified genes was not dependent on expression of c-Myb. The one example that we identified was the EPB41 gene, which has a c-Myb binding site identified by ChIP-on-chip and validated by conventional ChIP, although the gene was not induced by beta-estradiol stimulation (which leads to higher expression of c-Myb) and its expression did not decline when c-Myb expression was knocked down with an shRNA (Figure 4).

### A disconnect between c-myb RNA and c-Myb protein expression

The expression of c-*myb *RNA is affected by beta-estradiol stimulation, which relieves the transcriptional attenuation in intron I of the c-*myb *gene [6]. We found that beta-estradiol stimulation led to increased levels of c-*myb *RNA, although c-Myb protein levels remained constant and did not change accordingly. This unexpected result suggests that c-Myb protein levels may be regulated through a complex mechanism in MCF-7 cells. The c-Myb protein has sites of ubiquitinylation and sumoylation that can have a dramatic effect on its turnover and degradation rate [44-46]. Activation of Wnt signaling pathways can lead to phosphorylation, ubiquitinylation and degradation of c-Myb [47]. There is evidence of a link between the Wnt signalling and estrogen receptor pathways [48], and Wnt1 has been reported to be an estrogen receptor response gene [49], so it is possible that estrogen stimulation can simultaneously induce the expression of c-*myb *RNA and also lead to more rapid turnover and degradation of c-Myb protein, and that c-Myb protein could be stabilized in estrogen-deprived cells. This type of mechanism could explain why c-Myb protein levels appear to remain constant, even in estrogen-deprived MCF-7 cells.

### Estrogen stimulation affects c-Myb DNA binding to target genes

One of the most striking results from our study was the finding that c-Myb association with many target genes was greatly enhanced by beta-estradiol stimulation. This effect was seen both for endogenous c-Myb, and also for FLAG-tagged c-Myb expressed from a lentivirus vector (Figure 3). For example, enrichment of the CXCR4, JUN and CCNB1 promoters all increased at least 10-fold following beta-estradiol stimulation, suggesting that more c-Myb was associated with the promoters of these genes in the stimulated cells. These results could be explained by a lack of c-Myb binding to target gene promoters in estrogen-deprived cells. However, we found no significant change in c-Myb protein levels in the different growth conditions, so a change in binding would imply that c-Myb either failed to bind DNA or was relocated away from the active chromatin during estrogen depletion.

The c-Myb protein has not been reported to interact directly with Estrogen Receptors alpha or beta, although it does interact with a large collection of transcription factors and transcriptional co-activators [50], so there remains a possibility that the nuclear estrogen receptors could interact directly with c-Myb to affect its activity or to influence which genes c-Myb associates with. Our results suggest that c-Myb association with specific promoters is regulated and that c-Myb may associate with different subsets of target promoters in cells that are confluent compared to cells that are stimulated with beta-estradiol. This regulation could occur through changes in c-Myb protein, such as post-translational modifications, or changes in the expression or activity of proteins that cooperate with c-Myb at specific promoters. The results presented here provide the first evidence of large scale regulation of c-Myb activity and specificity in cells that are in different growth phases, suggesting that c-Myb is regulated largely by protein-protein interactions that help guide it to different target genes in different types of cells.

Estrogen treatment can lead to the activation of several signaling pathways through the alternative estrogen receptor GPER [51,52], so it is possible that these or other similarly-activated pathways are involved in the regulation of c-Myb DNA binding or localization during estrogen stimulation in MCF-7 cells. There is some evidence that the c-*myb *gene may be regulated by so-called non-genomic or alternative estrogen receptors [31]. Thus, changes in signal transduction pathways, perhaps regulated by these non-classical estrogen receptors, could lead to changes in c-Myb protein activity and/or specificity. Our results using ChIP assays provide the first evidence of large scale changes in c-Myb DNA binding activity occurring in response to cellular signals, and provide a new platform for studies of c-Myb regulation in future studies.

### Implications for the mechanisms of gene regulation by c-Myb

Our results raise the question of why c-Myb and other transcription factors are associated with so many promoters, especially since our own microarray studies only identified about 250 genes whose expression levels changed when c-Myb or v-Myb were over-expressed in MCF-7 cells [18,19]. One explanation could be the large number of intra- and inter-chromosomal interactions detected by long-range cross-linking methods [53], which detect interactions between promoters and distant regulatory elements. In some cases, a single promoter can make dozens of interactions with other parts of the chromosome, or even other chromosomes [54]. The ChIP-on-chip methodology we used only detects promoters, since only probes for the promoters are on the tiling arrays that we used. However, we could be detecting long-range interactions between c-Myb bound at distant sites interacting with the promoters that we detect. Recent findings showing that c-Myb binds both to promoters and distant enhancers supports this view [55], and implicates c-Myb in the regulation of long-range interactions between promoters and distant regulatory elements. The use of more advanced techniques will be required to resolve these issues and to understand which types of signals and regulators play a role in controlling the activity of c-Myb.

## Conclusions

Our results show that c-Myb associates with a surprisingly large number of promoters in human cells. The results also suggest that beta-estradiol stimulation leads to large-scale, genome-wide changes in c-Myb activity and subsequent changes in gene expression in human breast cancer cells. These results have important implications for understanding how signaling pathways affect the activity of transcription factors like c-Myb, and for understanding how stimulation of estrogen receptors, both classical and non-classical, affects so many genes in breast cancer cells. Our results suggest that c-Myb activity is dramatically different in cells that are in different growth conditions, and highlight the necessity for future studies to include characterization of both RNA levels as well as protein activity in order to unmask the role of c-Myb in cancer and normal cells.

## List of abbreviations

ChIP: Chromatin Immunoprecipitation; ChIP-on-chip: Chromatin Immunoprecipitation hybridized to a tiling array; ERs: estrogen receptors; IgG: immunoglobulin; kb: kilobase; QPCR: Quantitative real time PCR; shRNA: short hairpin RNA

## Competing interests

The authors declare that they have no competing interests.

## Authors' contributions

AMQ performed the ChIP and ChIP on chip, estrogen stimulation time course, gene expression assays associated with characterization of c-Myb binding and activity and drafted the manuscript. FL constructed the CXCR4 luciferase reporter and performed luciferase with c-Myb and v-Myb. JPO produced lentiviruses expressing FLAG c-Myb and v-Myb and shRNAs and transduced cells. SAN conceived of and supervised the project, analyzed the ChIP-on-chip data and wrote the final manuscript. All authors read and approved the final manuscript.

## Pre-publication history

The pre-publication history for this paper can be accessed here:

http://www.biomedcentral.com/1471-2407/11/30/prepub

## Supplementary Material

Additional file 1 **This file contains information about the custom and commercial primer sets used for QPCR assays (Tables S1, S2 and S3), and the locations and sequences of the putative Myb binding sites in the human CXCR4 gene promoter (Table S4)**. Also included is Figure S1, which diagrams the Myb binding sites identified in the ChiP-on-chip assays for several target genes, and Figure S2, which shows control Western blots confirming the expression of some of the transduced Myb proteins described in this study and the effects of the shRNA knockdowns, which is included as supplemental data since it confirms results previously published by others [6]. Click here for file

Additional file 2 **The ChIP-on-chip results obtained using confluent cells and antibody 1.1 (tab-delimited text file)**. Click here for file

Additional file 3 **The ChIP-on-chip results obtained using confluent cells and antibody 1493 (tab-delimited text file)**. Click here for file

Additional file 4 **The ChIP-on-chip results obtained using estrogen-deprived then beta-estradiol stimulated cells and antibody 1.1 (tab-delimited text file)**. Click here for file

Additional file 5 **The ChIP-on-chip results obtained using estrogen-deprived then beta-estradiol stimulated cells and antibody 1493 (tab-delimited text file)**. Click here for file

Additional file 6 **The merged ChIP-on-chip data with the gene annotations (tab-delimited text file)**. The raw ChIP on chip data has been deposited in the NCBI GEO database (GEO:GSE18706). Click here for file

## References

[B1] Gonda TJ Leo P Ramsay RG Estrogen and MYB in breast cancer: potential for new therapies Expert Opin Biol Ther 2008 8 713 717 10.1517/14712598.8.6.713 18476782

[B2] Guerin M Sheng ZM Andrieu N Riou G Strong association between c-myb and oestrogen-receptor expression in human breast cancer Oncogene 1990 5 131 135 2181374

[B3] Kauraniemi P Hedenfalk I Persson K Duggan DJ Tanner M Johannsson O Olsson H Trent JM Isola J Borg A MYB oncogene amplification in hereditary BRCA1 breast cancer Cancer Res 2000 60 5323 5328 11034064

[B4] Gudas JM Klein RC Oka M Cowan KH Posttranscriptional regulation of the c-myb proto-oncogene in estrogen receptor-positive breast cancer cells Clin Cancer Res 1995 1 235 243 9815978

[B5] Persson M Andren Y Mark J Horlings HM Persson F Stenman G Recurrent fusion of MYB and NFIB transcription factor genes in carcinomas of the breast and head and neck Proc Natl Acad Sci USA 2009 106 18740 18744 10.1073/pnas.0909114106 19841262 PMC2773970

[B6] Drabsch Y Hugo H Zhang R Dowhan DH Miao YR Gewirtz AM Barry SC Ramsay RG Gonda TJ Mechanism of and requirement for estrogen-regulated MYB expression in estrogen-receptor-positive breast cancer cells Proc Natl Acad Sci USA 2007 104 13762 13767 10.1073/pnas.0700104104 17690249 PMC1959456

[B7] Hodges LC Cook JD Lobenhofer EK Li L Bennett L Bushel PR Aldaz CM Afshari CA Walker CL Tamoxifen functions as a molecular agonist inducing cell cycle-associated genes in breast cancer cells Mol Cancer Res 2003 1 300 311 12612058

[B8] Cicatiello L Mutarelli M Grober OM Paris O Ferraro L Ravo M Tarallo R Luo S Schroth GP Seifert M Zinser C Chiusano ML Traini A De Bortoli M Weisz A Estrogen receptor alpha controls a gene network in luminal-like breast cancer cells comprising multiple transcription factors and microRNAs Am J Pathol 2010 176 2113 2130 10.2353/ajpath.2010.090837 20348243 PMC2861078

[B9] Jin W Liu Y Chen L Zhu H Di GH Ling H Wu J Shao ZM Involvement of MyoD and c-myb in regulation of basal and estrogen-induced transcription activity of the BRCA1 gene Breast Cancer Res Treat 2010 125 3 699 713 10.1007/s10549-010-0876-1 20364308

[B10] Chen L Xu S Zeng X Li J Yin W Chen Y Shao Z Jin W c-myb activates CXCL12 transcription in T47D and MCF7 breast cancer cells Acta Biochim Biophys Sin (Shanghai) 2010 42 1 7 10.1093/abbs/gmp108 20043041

[B11] Deisenroth C Thorner AR Enomoto T Perou CM Zhang Y Mitochondrial Hep27 is a c-Myb target gene that inhibits Mdm2 and stabilizes p53 Mol Cell Biol 2010 30 16 3981 93 10.1128/MCB.01284-09 20547751 PMC2916441

[B12] Ramsay RG Gonda TJ MYB function in normal and cancer cells Nat Rev Cancer 2008 8 523 534 10.1038/nrc2439 18574464

[B13] Mucenski ML McLain K Kier AB Swerdlow SH Schreiner CM Miller TA Pietryga DW Scott WJJ Potter SS A functional c-myb gene is required for normal murine fetal hepatic hematopoiesis Cell 1991 65 677 689 10.1016/0092-8674(91)90099-K 1709592

[B14] Malaterre J Carpinelli M Ernst M Alexander W Cooke M Sutton S Dworkin S Heath JK Frampton J McArthur G Clevers H Hilton D Mantamadiotis T Ramsay RG c-Myb is required for progenitor cell homeostasis in colonic crypts Proc Natl Acad Sci USA 2007 104 3829 3834 10.1073/pnas.0610055104 17360438 PMC1820669

[B15] Zorbas M Sicurella C Bertoncello I Venter D Ellis S Mucenski ML Ramsay RG c-Myb is critical for murine colon development Oncogene 1999 18 5821 5830 10.1038/sj.onc.1202971 10523863

[B16] Hugo H Cures A Suraweera N Drabsch Y Purcell D Mantamadiotis T Phillips W Dobrovic A Zupi G Gonda TJ Iacopetta B Ramsay RG Mutations in the MYB intron I regulatory sequence increase transcription in colon cancers Genes Chromosomes Cancer 2006 45 1143 1154 10.1002/gcc.20378 16977606

[B17] Greco C Alvino S Buglioni S Assisi D Lapenta R Grassi A Stigliano V Mottolese M Casale V Activation of c-MYC and c-MYB proto-oncogenes is associated with decreased apoptosis in tumor colon progression Anticancer Res 2001 21 3185 3192 11848471

[B18] Liu F Lei W O'Rourke JP Ness SA Oncogenic mutations cause dramatic, qualitative changes in the transcriptional activity of c-Myb Oncogene 2006 25 795 805 10.1038/sj.onc.1209105 16205643

[B19] Rushton JJ Davis LM Lei W Mo X Leutz A Ness SA Distinct changes in gene expression induced by A-Myb, B-Myb and c-Myb proteins Oncogene 2003 22 308 313 10.1038/sj.onc.1206131 12527900

[B20] Wells J Farnham PJ Characterizing transcription factor binding sites using formaldehyde crosslinking and immunoprecipitation Methods 2002 26 48 56 10.1016/S1046-2023(02)00007-5 12054904

[B21] Acevedo LG Iniguez AL Holster HL Zhang X Green R Farnham PJ Genome-scale ChIP-chip analysis using 10,000 human cells Biotechniques 2007 43 791 797 10.2144/000112625 18251256 PMC2268896

[B22] Yang J Chai L Fowles TC Alipio Z Xu D Fink LM Ward DC Ma Y Genome-wide analysis reveals Sall4 to be a major regulator of pluripotency in murine-embryonic stem cells Proc Natl Acad Sci USA 2008 105 19756 19761 10.1073/pnas.0809321105 19060217 PMC2604985

[B23] Zeller KI Zhao X Lee CW Chiu KP Yao F Yustein JT Ooi HS Orlov YL Shahab A Yong HC Fu Y Weng Z Kuznetsov VA Sung WK Ruan Y Dang CV Wei CL Global mapping of c-Myc binding sites and target gene networks in human B cells Proc Natl Acad Sci USA 2006 103 17834 17839 10.1073/pnas.0604129103 17093053 PMC1635161

[B24] Welboren WJ van Driel MA Janssen-Megens EM van Heeringen SJ Sweep FC Span PN Stunnenberg HG ChIP-Seq of ERalpha and RNA polymerase II defines genes differentially responding to ligands EMBO J 2009 28 1418 1428 10.1038/emboj.2009.88 19339991 PMC2688537

[B25] Kasowski M Grubert F Heffelfinger C Hariharan M Asabere A Waszak SM Habegger L Rozowsky J Shi M Urban AE Hong MY Karczewski KJ Huber W Weissman SM Gerstein MB Korbel JO Snyder M Variation in transcription factor binding among humans Science 2010 328 232 235 10.1126/science.1183621 20299548 PMC2938768

[B26] Lei W Liu F Ness SA Positive and negative regulation of c-Myb by cyclin D1, cyclin-dependent kinases, and p27 Kip1 Blood 2005 105 3855 3861 10.1182/blood-2004-08-3342 15687240 PMC1895079

[B27] O'Rourke JP Ness SA Alternative RNA splicing produces multiple forms of c-Myb with unique transcriptional activities Mol Cell Biol 2008 28 2091 2101 18195038 10.1128/MCB.01870-07 PMC2268396

[B28] Dash AB Orrico FC Ness SA The EVES motif mediates both intermolecular and intramolecular regulation of c-Myb Genes Dev 1996 10 1858 1869 10.1101/gad.10.15.1858 8756344

[B29] Johnson WE Li W Meyer CA Gottardo R Carroll JS Brown M Liu XS Model-based analysis of tiling-arrays for ChIP-chip Proc Natl Acad Sci USA 2006 103 12457 12462 10.1073/pnas.0601180103 16895995 PMC1567901

[B30] Klempnauer KH Bonifer C Sippel AE Identification and characterization of the protein encoded by the human c-myb proto-oncogene EMBO J 1986 5 1903 1911 3530745 10.1002/j.1460-2075.1986.tb04443.x PMC1167057

[B31] Jeng MH Shupnik MA Bender TP Westin EH Bandyopadhyay D Kumar R Masamura S Santen RJ Estrogen receptor expression and function in long-term estrogen-deprived human breast cancer cells Endocrinology 1998 139 4164 4174 10.1210/en.139.10.4164 9751496

[B32] Huang SC Cho A Norton S Liu ES Park J Zhou A Munagala ID Ou AC Yang G Wickrema A Tang TK Benz EJJ Coupled transcription-splicing regulation of mutually exclusive splicing events at the 5' exons of protein 4.1R gene Blood 2009 114 4233 4242 10.1182/blood-2009-02-206219 19729518 PMC2774555

[B33] Okumura K Mochizuki E Yokohama M Yamakawa H Shitara H Mburu P Yonekawa H Brown SD Kikkawa Y Protein 4.1 expression in the developing hair cells of the mouse inner ear Brain Res 2010 1307 53 62 10.1016/j.brainres.2009.10.039 19853587

[B34] Nakata Y Shetzline S Sakashita C Kalota A Rallapalli R Rudnick SI Zhang Y Emerson SG Gewirtz AM c-Myb contributes to G2/M cell cycle transition in human hematopoietic cells by direct regulation of cyclin B1 expression Mol Cell Biol 2007 27 2048 2058 10.1128/MCB.01100-06 17242210 PMC1820494

[B35] Kidder BL Yang J Palmer S Stat3 and c-Myc genome-wide promoter occupancy in embryonic stem cells PLoS One 2008 3 e3932 10.1371/journal.pone.0003932 19079543 PMC2592696

[B36] Wernig M Meissner A Foreman R Brambrink T Ku M Hochedlinger K Bernstein BE Jaenisch R In vitro reprogramming of fibroblasts into a pluripotent ES-cell-like state Nature 2007 448 318 324 10.1038/nature05944 17554336

[B37] Hall JM Korach KS Stromal cell-derived factor 1, a novel target of estrogen receptor action, mediates the mitogenic effects of estradiol in ovarian and breast cancer cells Mol Endocrinol 2003 17 792 803 10.1210/me.2002-0438 12586845

[B38] Hassan S Ferrario C Saragovi U Quenneville L Gaboury L Baccarelli A Salvucci O Basik M The influence of tumor-host interactions in the stromal cell-derived factor-1/CXCR4 ligand/receptor axis in determining metastatic risk in breast cancer Am J Pathol 2009 175 66 73 10.2353/ajpath.2009.080948 19497995 PMC2708795

[B39] Konoplev S Rassidakis GZ Estey E Kantarjian H Liakou CI Huang X Xiao L Andreeff M Konopleva M Medeiros LJ Overexpression of CXCR4 predicts adverse overall and event-free survival in patients with unmutated FLT3 acute myeloid leukemia with normal karyotype Cancer 2007 109 1152 1156 10.1002/cncr.22510 17315232

[B40] Spoo AC Lubbert M Wierda WG Burger JA CXCR4 is a prognostic marker in acute myelogenous leukemia Blood 2007 109 786 791 10.1182/blood-2006-05-024844 16888090

[B41] Tavor S Eisenbach M Jacob-Hirsch J Golan T Petit I Benzion K Kay S Baron S Amariglio N Deutsch V Naparstek E Rechavi G The CXCR4 antagonist AMD3100 impairs survival of human AML cells and induces their differentiation Leukemia 2008 22 2151 5158 10.1038/leu.2008.238 18769446

[B42] Caruz A Samsom M Alonso JM Alcami J Baleux F Virelizier JL Parmentier M Arenzana-Seisdedos F Genomic organization and promoter characterization of human CXCR4 gene FEBS Lett 1998 426 271 278 10.1016/S0014-5793(98)00359-7 9599023

[B43] Fan J Zeller K Chen YC Watkins T Barnes KC Becker KG Dang CV Cheadle C Time-dependent c-Myc transactomes mapped by Array-based nuclear run-on reveal transcriptional modules in human B cells PLoS One 2010 5 e9691 10.1371/journal.pone.0009691 20300622 PMC2837740

[B44] Bies J Feikova S Bottaro DP Wolff L Hyperphosphorylation and increased proteolytic breakdown of c-Myb induced by the inhibition of Ser/Thr protein phosphatases Oncogene 2000 19 2846 2854 10.1038/sj.onc.1203613 10851088

[B45] Dahle O Andersen TO Nordgard O Matre V Del Sal G Gabrielsen OS Transactivation properties of c-Myb are critically dependent on two SUMO-1 acceptor sites that are conjugated in a PIASy enhanced manner Eur J Biochem 2003 270 1338 1348 10.1046/j.1432-1033.2003.03504.x 12631292

[B46] Sramko M Markus J Kabat J Wolff L Bies J Stress-induced inactivation of the c-Myb transcription factor through conjugation of SUMO-2/3 proteins J Biol Chem 2006 281 40065 40075 10.1074/jbc.M609404200 17077080

[B47] Kanei-Ishii C Ninomiya-Tsuji J Tanikawa J Nomura T Ishitani T Kishida S Kokura K Kurahashi T Ichikawa-Iwata E Kim Y Matsumoto K Ishii S Wnt-1 signal induces phosphorylation and degradation of c-Myb protein via TAK1, HIPK2, and NLK Genes Dev 2004 18 816 829 10.1101/gad.1170604 15082531 PMC387421

[B48] Schlange T Matsuda Y Lienhard S Huber A Hynes NE Autocrine WNT signaling contributes to breast cancer cell proliferation via the canonical WNT pathway and EGFR transactivation Breast Cancer Res 2007 9 R63 10.1186/bcr1769 17897439 PMC2242658

[B49] Katoh M Expression and regulation of WNT1 in human cancer: up-regulation of WNT1 by beta-estradiol in MCF-7 cells Int J Oncol 2003 22 209 212 12469206

[B50] Ness SA Myb binding proteins: regulators and cohorts in transformation Oncogene 1999 18 3039 3046 10.1038/sj.onc.1202726 10378699

[B51] Filardo EJ Quinn JA Bland KI Frackelton ARJ Estrogen-induced activation of Erk-1 and Erk-2 requires the G protein-coupled receptor homolog, GPR30, and occurs via trans-activation of the epidermal growth factor receptor through release of HB-EGF Mol Endocrinol 2000 14 1649 1660 10.1210/me.14.10.1649 11043579

[B52] Revankar CM Cimino DF Sklar LA Arterburn JB Prossnitz ER A transmembrane intracellular estrogen receptor mediates rapid cell signaling Science 2005 307 1625 1630 10.1126/science.1106943 15705806

[B53] Lieberman-Aiden E van Berkum NL Williams L Imakaev M Ragoczy T Telling A Amit I Lajoie BR Sabo PJ Dorschner MO Sandstrom R Bernstein B Bender MA Groudine M Gnirke A Stamatoyannopoulos J Mirny LA Lander ES Dekker J Comprehensive mapping of long-range interactions reveals folding principles of the human genome Science 2009 326 289 293 10.1126/science.1181369 19815776 PMC2858594

[B54] Pink RC Eskiw CH Caley DP Carter DR Analysis of beta-globin chromatin micro- environment using a novel 3C variant, 4Cv PLoS One 2010 5 e13045 pii 10.1371/journal.pone.0013045 20927371 PMC2947503

[B55] Wilczek C Chayka O Plachetka A Klempnauer KH Myb-induced chromatin remodeling at a dual enhancer/promoter element involves non-coding rna transcription and is disrupted by oncogenic mutations of v-myb J Biol Chem 2009 284 35314 35324 10.1074/jbc.M109.066175 19841477 PMC2790961

