# Supplementary material for: Identification and Regulation of c-Myb Target Genes in MCF-7 Cells
Source: BMC Cancer. 2011 Jan 25;11:30. doi: 10.1186/1471-2407-11-30 (PMC3038977; doi:10.1186/1471-2407-11-30)

# Supplemental Information:

# Identification and Regulation of c-Myb Target Genes in MCF-7 Cells

Authors:

Anita M. Quintana, Fan Liu, John P. O’Rourke, and Scott A. Ness

Supplemental Methods

Detailed Chromatin Immunoprecipitation Assay Description

For chromatin immunoprecipitation, approximately 1x10^6^ cells were resuspended in 500 µl of cell lysis buffer (20 mM Tris-HCl pH 8.0, 85 mM KCl, 0.5% NP-40 plus protease inhibitors mix: 1 mM each of chymostatin, leupeptin, antipain and Pepstatin A and 1 mM each of Phenylmethanesulfonyl fluoride and benzamidine, all from Sigma-Aldrich, St. Louis MO), incubated on ice for 10 min then lysed by passing up and down 2x through a 26 G needle. The nuclei were recovered by centrifugation for 10 min at 14,000 RPM in the microcentrifuge and resuspended in 100 µl of 50 mM Tris-HCl pH 8.0 containing 5 mM CaCl2 plus protease inhibitors. The chromatin was sheared by adding 6 U of micrococcal nuclease (USB, Cleveland OH) for 10 min at 37°C, followed by adding EDTA to 10 mM and SDS to 1% and by heating to 65°C for 5 min to stop the reaction. The tubes were placed on a vortex mixer for 5 min at 4°C, then centrifuged 10 min at top speed in the microcentrifuge (14,000 RPM) to remove the debris. The supernatants containing chromatin fragments were recovered and diluted 10-fold with IP dilution buffer (0.01% SDS, 1.1% NP40, 1.2mM EDTA, 16.7 mM Tris-HCl pH 8.0, 167 mM NaCl plus protease inhibitors). Antibodies were added and incubated overnight. Immunoprecipitates were collected by adding protein A/G agarose (30 μl) (Santa Cruz Biotechnology, Santa Cruz, CA) for 30 minutes at 4°C. The agarose: antibody complexes were harvested by centrifugation and washed two times for 5 min each with low salt buffer (0.1% SDS, 1%Triton X-100, 2 mM EDTA, 10 mM Tris-HCl pH 8.0, 150 mM NaCl, plus protease inhibitors), high salt buffer (same as low salt buffer but 500 mM NaCl), LiCl2 buffer (10 mM Tris-HCl pH8.0, 250 mM LiCl, 1 mM EDTA, 1% NP40, 1% Deoxycholate plus protease inhibitors) and then TE (10 mM Tris-HCl pH 7.4, 1 mM EDTA plus protease inhibitors). The beads were resuspended in 100 µl of 1 % SDS plus protease inhibitors and incubated 5 min at 65°C to elute the immunoprecipitates. The samples were centrifuged and the supernatants were transferred to new tubes, NaCl was added to a final concentration of 0.3 M and then they were incubated at 65°C overnight to reverse the crosslinks. Then the samples were mixed with 2.5 volumes of cold ethanol, incubated at -20°C for 2 hr and centrifuged for 10 min in the microcentrifuge (14,000 RPM) to collect the DNA pellet, which was rinsed with 70% ethanol, re-centrifuged, drained and resuspended in 100 µl of 10 mM Tris-HCl pH 8.0. The samples were supplemented with 1 µl Proteinase K (10 mg/ml, Sigma-Aldrich) and 1 µl RNase A (100 µg/ml, Sigma-Aldrich) and incubated at 42°C for 2 hr. The final DNA sample was purified using a Qiagen MinElute PCR Purification Kit, following the manufacturer’s instructions, and samples of the final product were assayed by quantitative real-time PCR using gene-specific primer sets (Supplemental Table 2). For ChIP-on-chip assays, samples of the recovered DNA were amplified using primers with randomized ends, as described by Affymetrix. Samples were analyzed by real-time PCR before and after the amplification to ensure that linearity was maintained, since over-amplification resulted in loss of enrichment.

**Table S1: Expression Primers**

| **Primer** | **Forward** | **Reverse** |
| --- | --- | --- |
| KLF4 | ACGATCGTCTTCCCCTCTTT | ACCCTGGGTCTTGAGGAAGT |
| EPB41 | TGGCCCAAAGTGCTGAAGAT | AGTCCGGCCACTGTATCGAA |
| JUN | CCAATTCGCCCCACTATAAA | CTGGGAAACTCCTGAATTTGTAACT |

**Table S2: Taqman Expression Probes**

| **Primer** | **Catalog Number** |
| --- | --- |
| CXCR4 | HS00607978_s1 |
| c-myb | Hs00193527_m1 |
| CCNB1 | Hs00259126_m1 |
| PPIA | 4333763T |

**Table S3: ChIP primer pairs**

| **Primer** | **Forward** | **Reverse** |
| --- | --- | --- |
| NS-1 | ACCCACCAAACGGGACTCCATACTC | TCTCTTGACGGCGTGGAATGG |
| NS-2 | TGACATGGACTGCCTTGCATA | ACTGAGAAGCATGACGGACAAGTA |
| CXCR4 | CTGCACCTGACCCTAGTGATGCT | CCAGACCTGGGAATGCTACAGTAGA |
| GAPDH | GTGCAGGAGTTCATGACCAAC | CACCTGGGCTAGAGTACA |
| KLF4 | CGTGGGGGCTAAAGCTTGT | CAGAGGTGACTATTCAACCGCATA |
| EPB41 | CAGCGAGCAGAGGGTTAAATCTAT | ATGGCCTGCTGGTCAATCT |
| JUN | GGGGTCGTCTGGCTGCTCGTAGAA | CCAATTGCGCCCACTATAAA |
| CCNB1 | TGGTAACAAAGTCAGTGAAC | TATCAATGACTTTTCCAGTAG |

**Table S4:**

TFSCAN of NT_005058.14|Hs2_5215:c5485730-5483730 Homo sapiens chromosome 2 genomic contig (CXCR4 promoter)

| **Site No.** | **Start** | **End** | **Sequence** |
| --- | --- | --- | --- |
| 1 | 126 | 131 | CAACGC |
| 2 | 171 | 176 | TATCGG |
| 3 | 179 | 184 | TAAATG |
| 4 | 356 | 361 | TTACTG |
| 5 | 377 | 382 | CATCTG |
| 6 | 437 | 442 | TATCTG |
| 7 | 443 | 448 | CACCTG |
| 8 | 593 | 598 | CTACTG |
| 9 | 647 | 652 | CTACTG |
| 10 | 735 | 740 | CACCTG |

Potential Myb binding sites in the CXCR4 promoter were found using the EMBOSS tfscan program, using the consensus site YAACKG and allowing 1 mismatch.

Figure S1: ChIP on chip identifies c-Myb target genes.

ChIP on chip was performed with 1.1 and 1493 antibodies using chromatin from MCF-7 cells that were deprived of estrogen for 48 hr followed by 24 hr of estrogen stimulation or grown to high density. Histograms show the normalized (relative to input) signal and red bars identify the statistically significant (P<1x10^5^) binding sites. Genes diagrammed above the number line are on the positive strand, with transcription from left to right, and genes below the number line are in the opposite orientation. The images come from Integrated Genome Browser (www.affymetrix.com) and the number lines show nucleotide numbers in the March 2006 build (NCBI 36/hg18) of the human genome.

Figure S2: Over-expression and knockdown of c-Myb.

(A) Immunoprecipitation of FLAG c-Myb from transduced MCF-7 cells. Transduced MCF-7 cells were immunoprecipitated with anti-IgG or anti-FLAG agarose. Western blot was performed with anti-c-Myb antibodies. (B) Western blot of c-Myb knockdown. MCF-7 cells transduced with a lentivirus expressing doxycycline inducible scrambled or shRNA MYB were treated with doxycycline for 24 hours. Western blot was performed with anti-c-Myb and anti-beta-actin. (C) Expression of FLAG-tagged v-Myb. Immunoprecipitation with anti-IgG or anti-FLAG antibodies was performed on FLAG-v-Myb transduced MCF-7 cells. Western blot was developed with anti-Myb antibodies. The labels “Aby”, “NS” and “v-Myb” indicate the relative positions of the cross-reacting antibody, a non-specific background band and the v-Myb protein, respectively.

Figure S1


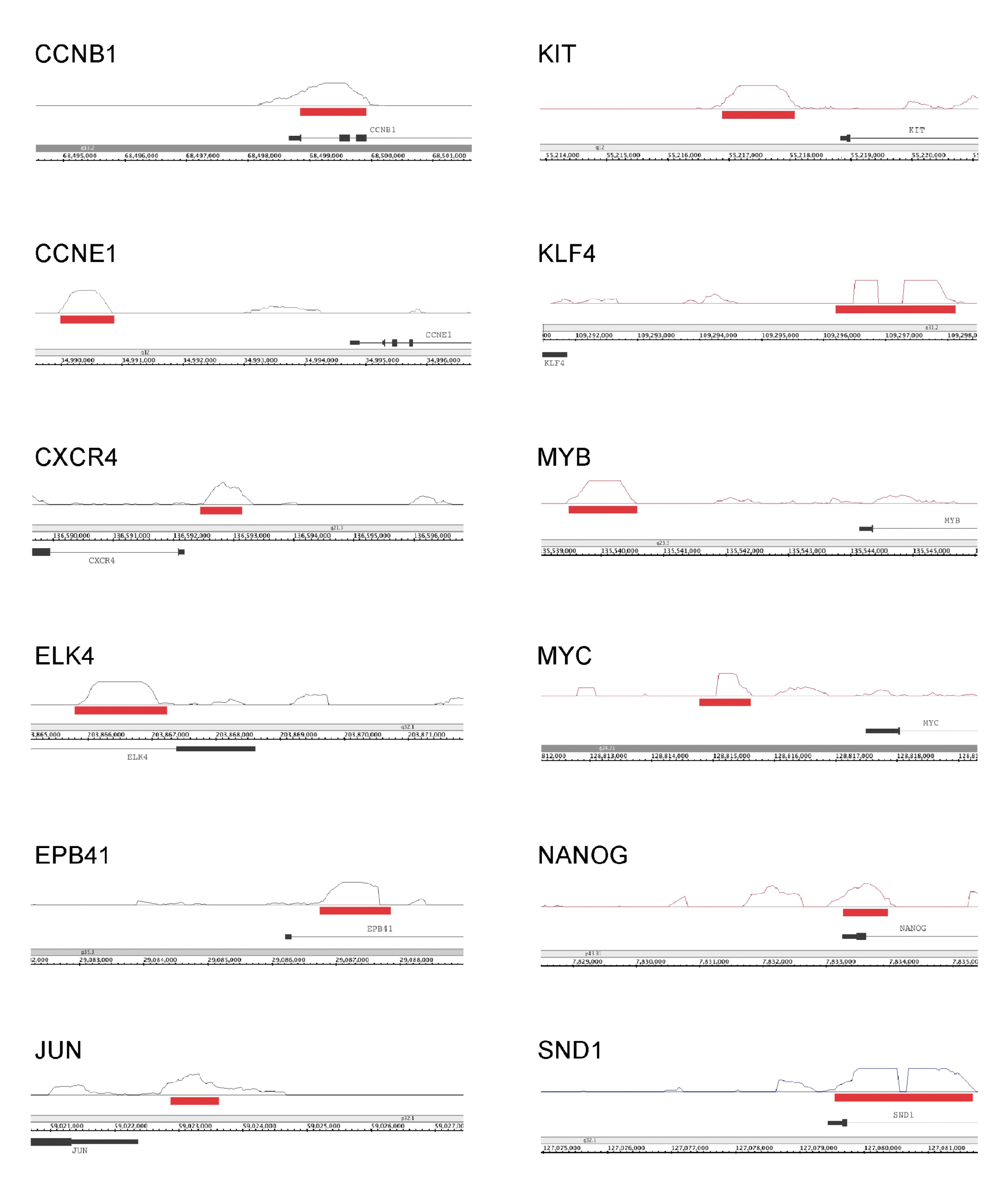


Figure S2


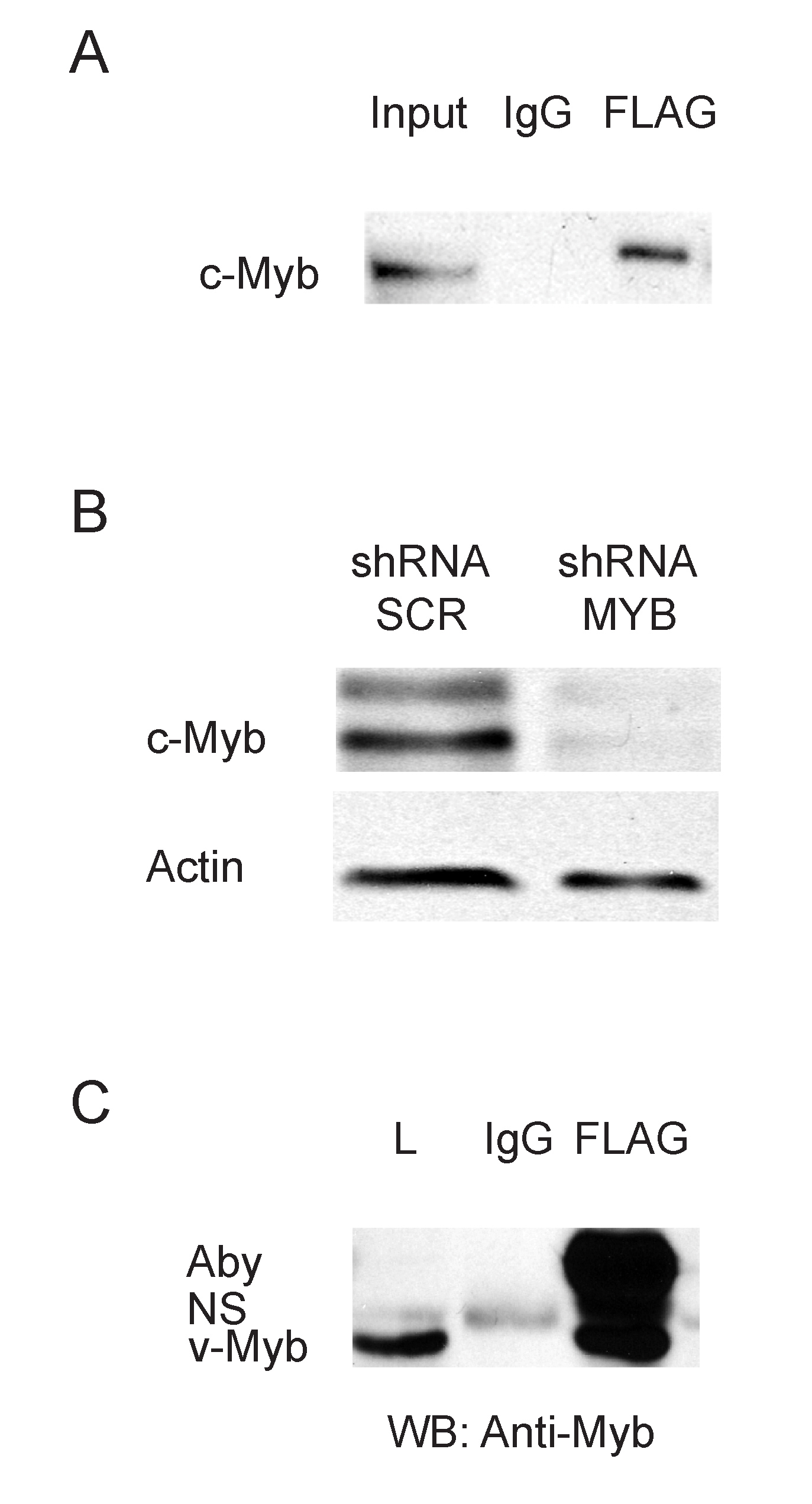

Supplement: Additional file 1 — This file contains information about the custom and commercial primer sets used for QPCR assays (Tables S1, S2 and S3), and the locations and sequences of the putative Myb binding sites in the human CXCR4 gene promoter (Table S4). Also included is Figure S1, which diagrams the Myb binding sites identified in the ChiP-on-chip assays for several target genes, and Figure S2, which shows control Western blots confirming the expression of some of the transduced Myb proteins described in this study and the effects of the shRNA knockdowns, which is included as supplemental data since it confirms results previously published by others [6]. [file 1471-2407-11-30-S1.DOC]
